# Advances in the development of hemostatic biomaterials for medical application

**DOI:** 10.1186/s40824-021-00239-1

**Published:** 2021-11-12

**Authors:** Yong Kiel Sung, Dae Ryeong Lee, Dong June Chung

**Affiliations:** 1 Department of Chemistry, College of Science, Dogguk University, Phil-dong, Seoul, South Korea; 2 Department of Polymer Science and Engineering, Sungkyunkwan University, Suwon, South Korea

**Keywords:** Hemostasis, Biomaterials, Blood clotting, Coagulation, Wound healing

## Abstract

**Background:**

Medical hemostatic biological materials are necessary for the development of the process of preventing and stopping damaged intravascular bleeding. In the process, some red blood cells and white blood cells are trapped in nets. The resulting plug is called a blood clot. This is often a good step in wound healing, but separation of blood clots from blood vessel walls can cause serious health problems.

**Main body:**

The advance in the development of hemostatic biomaterials is necessary for biomedical application. Firstly, the historical background of artificial hemostasis has been included and the current research of hemostasis has been included in more detail. Secondly, the current research of hemostasis has been included on the oxidized cellulose-based hemostatic biomaterials such as starch based on composite cross-linking hemostatic networks, hemostatic materials on *N*HS-esters, hemostatic agent from local materials and biomaterials for hemostatic management. Thirdly, polysaccharide hemostatic materials, bio-inspired adhesive catechol-conjugated chitosan, mesoporous silica and bioactive glasses for improved hemostasis, minimally invasive hemostatic biomaterials and chitosan-base materials for hemostatic application have been included. Fourthly, the biological properties of natural hemostatic agent by plasma technology and the hemostatic agents based on gelatin-microbial transglutaminase mixes have been also included.

**Conclusion:**

Current research on hemostasis includes hemostatic biomaterials such as cellulose-based hemostatic starch based on a complex cross-linked hemostatic network. It also includes polysaccharide hemostatic materials, biomimetic adhesive catechol-binding chitosan, mesoporous silica or physiologically active glass for hemostatic improvement, minimally invasive hemostatic chitosan-based materials, and gelatin-microbial transglutaminase-based hemostatic agents. Future studies should focus on modular combination of hemostatic imitation and reinforcement mechanisms of different materials and technologies to find the optimal system to promote and strengthen active platelet or platelet imitation aggregation in bleeding sites. The second optionally increases the production of thrombin and fiber formation at the site. Third, the formed fibrin biopolymer network has strengthened to reduce thrombosis and amplify hemostasis.

## Introduction

Hemostasis is a process that prevents and stops bleeding, and means maintaining blood with damaged blood vessels. This is the first step in wound healing and the blood clotting process. This includes coagulation, changing blood from liquid to hydrogel. Complete blood vessels are central to controlling blood trends that form blood clots. Undamaged blood vessels’ endothelial cells prevent blood clotting with heparin-like molecules and prevent platelet aggregation with nitrogen oxide [1, 2] and prostacycline [3]. When endothelial damage occurs, endothelial cells stop the secretion of coagulation and cohesion inhibitors and instead secrete vWF (von Willebrand factor) [4, 5], which starts maintaining hemostasis after damage. There are three main steps to hemostasis. i) Vascular contraction, ii) temporary blocking by platelet stopper, iii) blood clotting, iv) fibrin clot formation. Hemostasis is maintained in the body through the following four mechanisms.

i) Vascular contraction (vascular cramps): Vascular contraction is produced in vascular smooth muscle cells and is the first response of blood vessels to damage. Smooth muscle cells are controlled by vascular endothelial skin that emits intravascular signals to control shrinkage characteristics. Damage to blood vessels causes immediate reflection initiated by local sympathetic nerve pain receptors, promoting vascular contraction. Damaged blood vessels contract, reducing blood flow through the area and limiting the amount of blood loss. Collagen is exposed to the damaged area, and collagen promotes platelets to adhere to the damaged area. Platelets release cytoplasmic granules containing serotonin, ADP, and thrombocyte A2, all of which increase the vasoconstriction effect. The more the damage increases, the more effective the convulsions reaction is. Vascular spasms are much more effective in small blood vessels [6, 7].

ii) Platelet aggregation: platelet-rich human plasma is a turbid liquid. Adding adenosine diphosphate (ADP) activates and begins to agglomerate platelets to form white flakes. Hemostasis occurs when blood is outside the body or blood vessels. Stopping bleeding and blood loss is the body’s natural reaction. During hemostasis, three stages occur in a fast order. Vascular spasm is the first reaction in which blood vessels contract to reduce blood loss. In the second step, platelet stopper formation, platelets adhere to each other and form a temporary seal to cover gaps in the blood vessel wall. The third step is coagulation or blood coagulation. Coagulation strengthens the platelet plug with fibroblasts acting as molecular adhesives [8]. Platelets are a big factor in the hemostasis process. They allow the production of platelet caps that form immediately after a blood vessel rupture. Within a few seconds of destruction of the vascular epithelial wall, platelets begin to adhere to the surface under the endothelial skin. It takes about 60 s for the first fibrous strand to begin to scatter between the wounds. After a few minutes, the platelet stopper was completely formed [9].

iii) Platelet stopper formation; Platelet is attached to the damaged endothelial skin to form platelet stopper (primary hemostasis) and then degranulated. This process was controlled through thrombosis control. Plug formation is activated by a glycoprotein called the von Villebrandt factor found in plasma. Platelets play one of the important roles in the hemostasis process. Platelets change shape when they meet damaged endothelial cells, release granules, and ultimately become sticky. Platelets express certain receptors, some of which have been used to attach platelets to collagen. When platelets are activated, glycol protein receptors that interact with other platelets are expressed to produce aggregation and adhesion. Platelets emit cytoplasmic granules such as adenosine diphosphate (ADP), serotonin, and thromboxylic acid A2. Adenosine diphosphate attracts more platelets to the affected area, and thromboxylic acid A2 helps platelet aggregation, vascular contraction, and degranulation. Only platelets are responsible for preventing bleeding from invisible wear on our skin, so called primary hemostasis [10].

iv) Fibrin clot formation: Once the platelet plug having formed by the platelets, the clotting factors are activated in a sequence of events known as coagulation cascade, which leads to the formation of fibrin from inactive fibrinogen plasma protein. A fibrin mesh has produced all around the platelet plug to hold it in place; this step has called secondary hemostasis. During this process, some red and white blood cells have trapped in the mesh, which causes the primary hemostasis plugged to become harder. The resultant plug has called as thrombus or clot. The blood clot contains secondary hemostasis plug with blood cells trapped in it. Though that is often a good step for wound healing, it has the ability to cause severe health problems if the thrombus becomes detached from the vessel wall and travels through the circulatory system.

In the present paper, the recent advances in the development of hemostatic materials have been reviewed for biomedical applications. The historical background of artificial hemostasis has been described, and the current research of hemostasis has been also included in more detail introducing oxidized cellulose-based hemostatic materials, starch based composite cross-linking hemostatic networks, and the hemostatic materials. The current research of hemostasis has been also included about the oxidized cellulose-based hemostatic biomaterials such as starch based composite cross-linking hemostatic networks, hemostatic materials based on NHS-esters, hemostatic agent from local materials and biomaterials for hemostatic management. Polysaccharide hemostatic materials, bio-inspired adhesive catechol-conjugated chitosan, mesoporous silica and bioactive glasses for improved hemostasis, minimally invasive hemostatic biomaterials and chitosan-based materials for hemostatic application have been included. The biological properties of natural hemostatic agent by plasma technology and the hemostatic agents based on gelatin-microbial transglutaminase mixes have been also reviewed.

## Artificial hemostasis and current research of hemostasis

### Artificial hemostasis

The process of preventing blood loss in blood vessels or organs in the body is called hemostasis. The term comes from the Greek word hem, which means blood, and stasis, which means suspension. Putting it together means that blood stops [8]. Excessive bleeding inevitably started with the perception that it was like death. The Greeks and Romans used vegetable and mineral hemostatic agents for major wounds. At that time, Egypt’s mummification study led to much more progress in the general medical field, which led to more knowledge of the hemostasis process. During this period, many veins and arteries flowing throughout the body were found and the direction of movement was found. Doctors of this era realized that blood could not continue to flow out of the body if it was blocked. Nevertheless, until the invention of the printing press in the fifteenth century, medical records and ideas moved westward, enabling ideas and practices for hemostasis [11].

Local hemostatic devices control bleeding stably and quickly. Preferred formulations will be easy to store and manufacture at room temperature. It can be used immediately in the operating room and in various procedures in the operating room. In addition, the selected drugs will demonstrate safety and be inexpensive by minimizing blood transfusion [12]. In general, local hemostatic devices are classified into four categories: mechanical hemostatic devices, active hemostatic devices, fluid hemostatic devices, and fiber sealants, as shown in

**Table 1:**
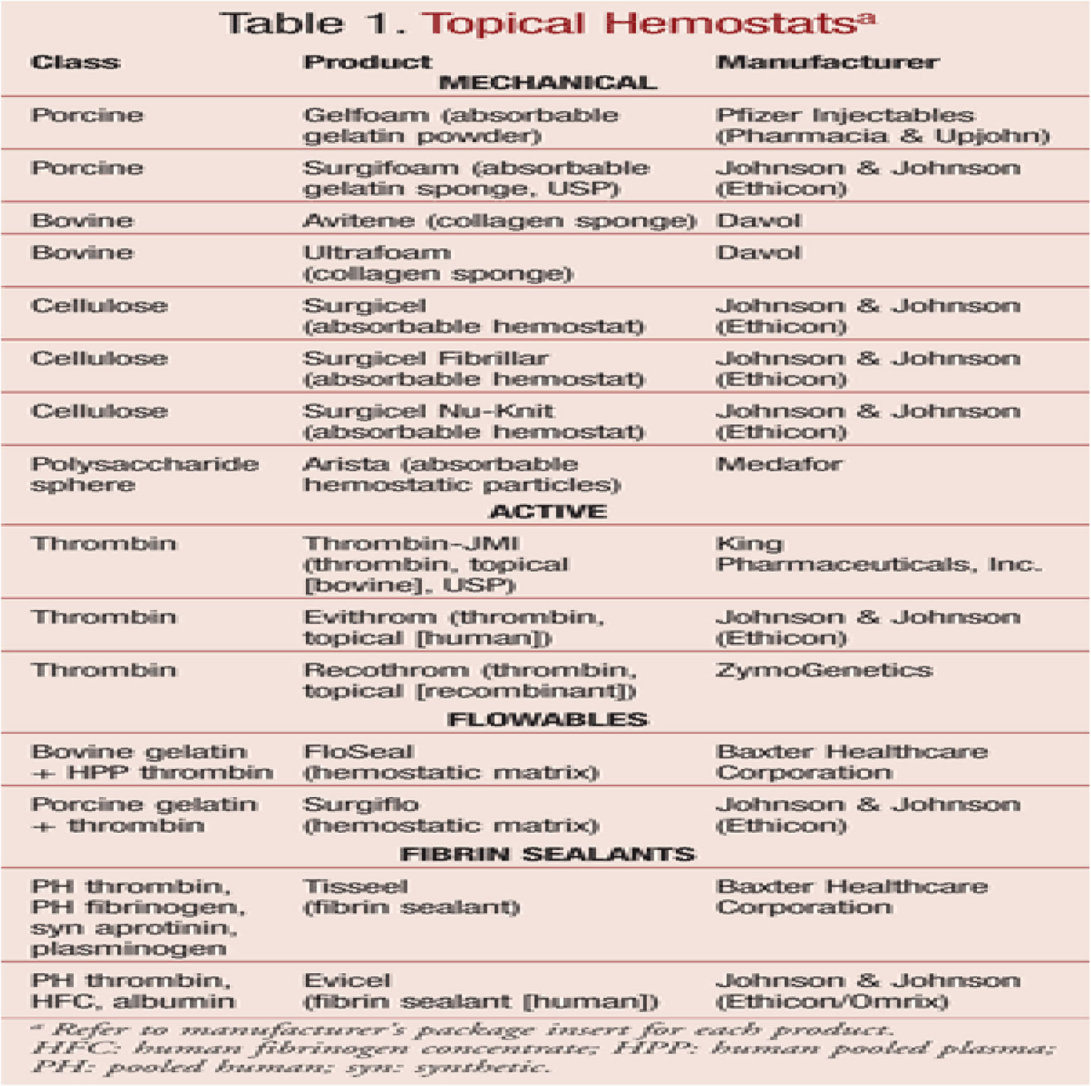
Topical Hemostats^a^. (a: References: 12 and 13).

Table 1 [12, 13].

### Mechanical hemostats

Mechanical hemostatic products are gelatin, collagen, cellulose and polysaccharide derivatives. Local mechanical hemostatic agents are applied as sponges and do not contain thrombin or other active biological compounds. They cause swelling and create mechanical barriers to bleeding. Products can cause abscess formation. Mechanical products are widely available in the operating room and are generally considered primary drugs because they are the cheapest local hemostatic agents [14]. .Examples of pig gelatin products include Gelfoam (Pfizer) and Surgifoam (Johnson & Johnson, Ethicon). Gelfoam products were used in all surgical procedures. Gelfoam Plus requires needles to prepare and contains pooled human thrombin. Therefore, there is a risk of spreading the virus. Gelfoam Plus’s trombin concentration is 125 IU/mL lower than other trombin products, which are 800 to 1200 IU/mL. Surgifoam has been used in all surgical procedures, but is not suitable for ophthalmic procedures [14]. .Socolagen products include Avitene (Davol) and Ultrafoam (Davol). Trombin does not need to be used with Ultrafoam sponge. Avitene is served in flour and sheets. Flour can stick to gloves and surgical instruments. Surge Cell, Surge Cell fibrillar, and Surge Cell Nu-Knit (all Johnson & Johnson) are examples of cellulose oxide products. Because thrombin is destroyed by the low pH of the product, the hemostatic effect of surge cells does not improve with the addition of thrombin. Arista (Medfor) is a plant-derived polysaccharide starch that acts like a body that dehydrates blood. These polysaccharide spheres concentrate the solid ingredients in the blood to help blood coagulation proteins.

### Active hemostats

Trombin is a proteolytic enzyme that converts fiber sources into fiber. Local thrombin has a direct coagulation effect on exposed blood. Active hemostatic agents include local (small) thrombin (Thrombin-JMI), local (human) thrombin (Evithrom), and local (recombination) thrombin (Recothrom). The drug indicated that it helps hemostasis whenever blood and minor bleeding from capillaries and small cleaning veins are accessible. The first clinical use of topical sotrombin dates back more than 60 years. FDA approval for Trombin was surprise in the 1970s. The use of topical thrombin has been included in more than 100 applications, including spine, nerve, general, orthopedic, heart, chest, blood vessels, gynecology, and dental procedures [15, 16]. Thrombin-JMI (King Pharmaceuticals) was approved in 1995 [17]. It is estimated that between 500,000 and 1 million patients are exposed to sotrombin on an annual basis in the United States. As a broad indication of its use, sotrombin has two sizes of vials and can be sprayed or applied to a sponge. In 1996, a black box warning was added to all sotrombin formulations. This warning indicates that it is sometimes associated with bovine thrombin and coagulation disorders. These coagulation disorders range from mild laboratory abnormalities (prothrombin time change [PT], partial thromboplastin time [PTT]) to mild bleeding (nonbleeding or hematoma), and severe bleeding (continuous and uncontrolled and life-threatening). This response is associated with antibodies to sotrombin and/or factor V that cross-react with human coagulation factors [18]. Factor V is an essential secondary factor in converting prothrombin into trombin. Factor V inhibitors can cause deactivation or depletion of factor V. The actual incidence of sotrombin-related coagulation is unknown for initial exposure or re-exposure. However, re-exposure to sotrombin increases the risk of antibody formation. The clinical features of immunomodized coagulopathy (IMC) vary widely. Antigen exposure is not documented in medical records [19]. The reported clinical manifestation time of IMC varies widely and can occur within an average of 32 to 10 days after exposure. Bleeding occurred in about half of these cases. Currently, clinical awareness of IMC is low [20–22]. .Newly formulated bovine-derived thrombin-JMI significantly reduced the level of pollutants, but coagulation disorders can still occur [22]. Hemorrhagic IMC is a diagnostic challenge. Clinical features can be highly variable and obscured by other conditions, such as anticoagulants or antiplatelet therapy, vitamin K or liver disease, disseminated intravascular coagulation, acid and hypothermia, and blood dilution due to blood loss [23, 24]. .Hemorrhagic patients may require red blood cell transfusion, platelet transfusion, and immunosuppressive therapy (corticosteroid, cyclosporin), chemotherapy (cyclophosphamide, vincristin), or epsilon aminocapric acid [25–27].

The FDA approved a pooled source of human plasma thrombin (Evithrom [Johnson & Johnson]) in 2007. The final product goes through virus inactivation to ensure the safety of the product. There is no black box warning in this product, but there is a risk of spreading the virus. Freeze you need to avoid thawing time before the administration and is evithrom Recombinant [ZymoGenetics] was approved by the FDA in 2008. This product is produced using recombinant DNA and has similar effects to sotrombin, but has no cow or human plasma. It was produced through the Chinese hamster ovary (CHO) host cell protein [28]. .Examples of other products produced through recombinant technology include human insulin, growth hormone, etanusept (enbrel), bevacizumab (abastin), and rituximab (lituxane) [29–31].

### Fluid hemostats

Fluid hemostat is thick but has a fluid consistency and contains a bovine or porcine gelatin matrix. This is useful when applying to hard-to-reach surfaces or wet fields. They can be used in all surgeries except ophthalmology. Surgiflo (Johnson & Johnson) contains porcine gelatin and is miscible with thrombin [32]. FloSeal (Baxter) contains bovine gelatin matrix and human pool plasma thrombin [33].

### Fibrin sealants

Fibrin sealant contains thrombin and fibrinogen and is useful when hemostatic agents and sealant are needed. Tissel (Baxter) contains pooled human thrombin, pooled human fibrinogen, synthetic aphrotinine, and plasminogen [34]. .Human thrombin is at risk of viral transmission and the product takes a long time to prepare. Tissel is the first surgical sealant approved by Evicel (Johnson & Johnson) in the United States in 1998. It contains human thrombin, human fibrinogen concentrate, and albumin. There is also a risk of spreading the virus. This product is for freezing and needs to be thawed [35].

## Current research on hemostatic biomaterials

Currently, many studies on hemostasis are being conducted. The most recent researches are based on the genetic factors of hemostasis. It has been changed to reduce the cause of genetic disorders that change the natural process of hemostasis [36]. Phonvillebrandt’s disease is ultimately associated with platelet caps that stop bleeding and defects in physical ability to produce fibroblasts. Recent studies have concluded that von Villebrandt disease is much more common in adolescence. This disease negatively interferes with the natural process of hemostasis, causing patients suffering from this disease to worry about excessive bleeding. There are complex treatments performed, including combinations of therapies, estrogen-progesterone formulations, desmopressin, and von Villebrandt factor concentrates. Currently, research is trying to find a better way to deal with diseases [36, 37].

### Cellulose oxide-based hemostatic materials

The application of hemostatic agents is essential to prevent serious bleeding and death from excessive bleeding in surgery or emergency situations. Cellulose oxide is an excellent biodegradable and biocompatible derivative of cellulose and has become one of the most important hemostatic agents used in surgical procedures. There is no comprehensive report on evaluating cellulose-based hemostatic substances. Preparation of oxidation, the origin and structure of cellulose, and the biodegradability and safety of cellulose oxide were reviewed. A comprehensive discussion of hemostatic mechanisms, various forms, variations, and currently commercially available cellulose products has been included, which highlights the most important development in recent scientific literature [37, 38].

### Starch based composite cross-linking hemostatic networks

Porous complex starch (PCS) with starch-based complex crosslinked hemostatic network chitosan (CS) was crosslinked by STMP (sodium trietaphosphate) to obtain hemostatic agents called STMP/PCS/CS (SPC) with ideal hemostatic effects. The absorption rate (WAR) and swelling rate (SR) of SPC reach 150.8% (WAR) and 355.0% (SR), respectively. It was observed by scanning electron microscope (SEM) that a large amount of CS was combined with PS, indicating that the composite effect was ideal. The elevated hemostasis effect of SPC was shown in blood cell evaluation experiments, and platelet activation triggers a multistage cascade that adsorbs more blood cells onto the SPC surface to produce thrombosis. In tail amputation 1 cm, 2 cm, and liver laceration, the average hemostasis time of SPC was reduced by 29, 42, and 37%, respectively, compared to the gap control group, and the hemostasis effect was better than that of CS and PS. Performance for rat tail cutting and liver lacerations [38, 39].

Chitosan is a powerful hemostatic agent that induces blood clotting even with extensive anticoagulant therapy. Blood coagulation has been suggested to be related to the possible formation of a polymer electrolyte complex (PEC) containing a negatively charged acidic group present on the surface of chitosan amino function and red blood cells. In contrast, chitin exhibits increased anticoagulant properties with O-sulfonation because it is similar to heparin, a naturally occurring glycosaminoglycan (GAG) used as an anticoagulant in clinical practice.

### NHS-Ester-based hemostatic ingredients

Hemostatic agents have been developed to prevent bleeding during surgery. Hemostatic devices using physiologically active ingredients to promote coagulation rely on natural sources that limit reproducibility. Hybrid devices using chain-terminated reactive poly (ethylene glycol) (PEG) as an active component may undergo irregular crosslinking and dissolution of polar PEG when blood flow is significant. Here they describe synthesized, non-physiologically active hemostatic products by coating N-hydroxysuccinimide (NHS) ester-functional poly (POx) on gelatin patches acting by covalent crosslinking between polymer and host blood. Protein, gelatin, and tissue seal wounds and prevent bleeding during surgery. They studied various process parameters, including polymers, carriers, and coating techniques, in direct comparison with clinical products (hemopatch and Tachosil), to gain a deeper understanding of these kinds of hemostatic products. They successfully demonstrated the hemostatic efficacy of POx-NHS as a polymer powder and coated patch in vitro and vivo against Hemopatch and Tachosil, demonstrating that POx-NHS is an excellent candidate polymer for hemostatic patches [39, 40].

### Hemostatic agent from local ingredients

Hemostatic agents are medical substances used in surgery to stop or control excessive bleeding. This study aimed to develop hemostatic agents produced from natural sources. The advantages of using natural materials are low cost, non-toxic, and reasonable decomposition. The local materials used in the study are rice starch and chitosan synthesized from squid pens. Both materials are biocompatible and are generally used in biomedical applications. Chitosan with antibacterial properties can be used as a hemostatic agent to prevent wound infection. Freeze drying was used to prepare hemostatic agents. An experimental design was implemented to optimize the ratio of rice starch to chitosan to produce hemostatic agents with appropriate blood absorption and expansion properties. As a result, chitosan and 50:50 hemostatic agents; rice starch volume ratio showed reasonable properties for bleeding control due to acceptable physical properties, fast blood absorption and low hemoglobin leakage. In addition, the hemostatic agent under development has low production costs and little superior properties compared to commercial products, so it is likely to be applied to medical applications [40, 41].

### Biological ingredients for hemostasis management

Bleeding complications caused by trauma, surgery and congenital, disease-related, or drug-induced blood disorders can cause serious morbidity and mortality in civilians and soldiers. Interruption of hemostasis is clinically most important in prevention, surgery, and emergency scenarios. In the case of externally accessible injuries, various natural and synthetic biomaterials went through strong research and led to hemostatic technologies including adhesives, bandages, tamponades, hemostatic bands, dressing and coagulation promoting powders. On the other hand, the treatment of incompressible internal bleeding still relies heavily on hemostatic components of blood, such as whole blood transfusions, platelets, fibrinogen, and coagulation factors. Platelet transfusions pose significant problems such as limited availability, high cost, pollution risk, low portability, performance variability, and immunological effects, while the use of fibrinogen or coagulation factors provides only partial mechanisms for hemostasis. Considering these points, many interdisciplinary research efforts have been focused on material and technology development. They can be conveniently included, sterilized to minimize contamination, and intravenously administered to imitate, utilize, and amplify physiological hemostasis mechanisms. They provide a comprehensive review of various local, intraperitoneal and intravenous hemostatic techniques in terms of materials, mechanisms and state-of-the-art technology [41, 42].

### Polysaccharide hemostatic agents

Stable thrombus formation or hemostasis is essential to prevent major bleeding and death from excessive bleeding. The coagulation process of the body itself cannot be stopped in a timely manner without the help of hemostatic agents. To develop new local hemostatic agents, tissue adhesives, and sealants, it is necessary to understand the coagulation process and hemostatic mechanisms of different materials. Polysaccharides among hemostatic substances are naturally derived polymers and have excellent biodegradability and biocompatibility. This article provides an overview of polysaccharide-based hemostatic materials and preparations, including advantages and disadvantages of hemostatic applications. Polysaccharide-based hemostatic substances with antibacterial and healing functions were included [42, 43]

### Biomimic adhesion catechol binding chitosan

The development of adhesive materials such as cyanoacrylate derivatives, fibrin adhesives and gelatin-based adhesives has become a new topic in biomaterials because these materials are widely used, including wound healing patches, tissue sealants and hemostatic materials. Most biological adhesives have poor adhesion to tissues and related surfaces due to the presence of body fluids. This study aimed to solve this problem by developing moisture-resistant adhesives. Mussels exhibit strong moisture-resistant adhesion despite constant waves on the beach, and mussels adhesive proteins play an important role in this adhesion. The adhesive protein located at the end consists of about 60% of amino acids called 3,4-dihydroxy-1-phenylalanine, lysine, and histidine, and has side chains of catechol, primary amine, and secondary amine, respectively. Inspired by catecholamine rich in mussel adhesive proteins, researchers have developed various types of polymer imitations such as poly (ethyleneimine)-catechol, chitosan-catechol, and other related catechol polymers. Chitosan-catechol is a promising adhesive polymer in the biomedical field. When catechol is bound to chitosan, the solubility in the pH 7 aqueous solution increases rapidly from 0 to almost 60 mg/mL. Improved solubility maximizes the ability of catecholamine to behave in similar mussel adhesive proteins. Chitosan-catechol is biocompatible and exhibits excellent hemostatic ability and tissue adhesion, and thus will be widely used in various biomedical environments for medical applications [43, 44].

### Mesoporous silica and physiologically active glass for improved hemostasis

Well-organized mesoporous silica and physiologically active glass of minerals have recently shown great potential to accelerate hemostasis and infection control. Immediate control of uncontrolled bleeding and infection is essential for saving lives both in combat and civilian. However, to date, there are no comprehensive reports evaluating specific mechanisms of action that accelerate the hemostasis process and exert antibacterial effects. After providing a brief review of hemostasis processes, the study presents a critical overview of the recently developed inorganic mesoporous silica and physiologically active glass-based materials for hemostasis clinical application. Their unique characteristics have been found to be applicable to hemostasis and infection prevention. This article also identifies promising new research directions initiated to identify the effectiveness of these materials for hemostasis application [44, 45].

### Chitosan-based materials for hemostatic application

Effective and rapid hemostasis is critical to surgery and emergency trauma, especially trauma occurring in battlefields and other complex situations [45, 46]. Hemostasis is an important step in emergency medical care. Effective hemostasis is essential to reducing patient pain and mortality, and research and development of hemostatic materials is a prerequisite for effective hemostasis. Chitosan, which is highly biocompatible and non-toxic, is widely applied to biopharmaceuticals, chemical industry, food industry, cosmetics, etc. [47]. The chitosan complex hemostatic material is currently emerging as a hemostatic material. After briefly introducing the hemostatic mechanism of chitosan, progress of research on chitosan-based composite hemostatic materials having various forms such as films, sponges, hydrogels, particles, and fibers was introduced. Chitosan-based complex hemostatic materials are perspectives on effective hemostatic materials in future studies. Chitosan nanoparticles have become famous for their biodegradability, easy availability, cancer imaging, and low cytotoxicity [48].

### Minimal invasive hemostatic biomaterials

Surgeons often experience internal bleeding in minimally invasive surgery (MIS). MIS is a surgical treatment method that minimizes trauma using laparoscopy, nasal endoscopy, and other medical equipment [49, 50]. Hemostasis in vivo is the key to success in minimally invasive surgery. Solid hemostatic materials cannot pass through the synth tube of the MIS device, so the tissue peels off. To address the adhesive dilemma, formulations containing multifunctional sucrose allyl ether (SAE) monomers and hydroxy-ketone photo-initiators have been adopted as lead hemostatic materials for MIS. Here, in vivo hemostasis experiments were performed by comparing the formulation with chitosan [51].

### Biological properties of natural hemostatic agent by plasma technology

Excessive bleeding is an important problem in surgery. It is a concern about bleeding during clinical procedures. Bleeding control and rapid blood coagulation are the main management tasks of surgery [51]. The purpose of the study was to evaluate the effectiveness of non-plasma treatment on the biological properties of natural hemostatic agents. Studies have shown that plasma treatment can increase the decomposition rate of samples by up to 94.26% within 7 days, improving the biodegradability of hemostatic agents. In addition, the plasma-treated sample showed excellent biocompatibility in the cell survival rate test of fibroblasts. Cell growth and cell proliferation in this sample were found to be helpful in the wound healing process. Modified drugs can help better control bleeding during surgery to develop new hemostatic products that meet the requirements, including biomedical applications with proper biocompatibility and biodegradability [52].

### Gelatin-microbial transglutaminase mixture-based hemostatic agents

Effective and rapid hemostasis is important to increase post-traumatic survival. Ideal hemostatic agents should have performance characteristics such as rapid hemostatic effect and simple preparation for various types of bleeding and be non-immune [53–55]. Existing hemostasis methods have limitations in efficacy and can cause additional tissue damage. This study designed a new hemostatic agent based on the formation of in situ gels in gelatin, in which amino acid sequences are crosslinked by new microbial transglutaminases different from commercial mTGases. The new hemostatic agent exhibits the same biochemical crosslinking effect as the final stage of the blood coagulation cascade while using gelatin as a structural protein (instead of fibrin) and calcium-dependent mTGase as a crosslinking catalyst (instead of factor XIIIa). Biomimetic gelatin-mTGase mixed hemostatic agents are effective surgical sealers [56]. In the liver hemostasis model of mice, hemostatic agents showed similar hemostatic effects to SURGIFLO®, as well as stronger adhesion and elasticity than SURGIFLO®.

### Blood interactions with micro- and nano-fibers

Fine and nanofiber materials seek a wide range of applications, such as vascular grafts, tissue engineering scaffolds, and medical drug delivery systems. These applications are due to surface functionalization methods, as well as almost any biomaterial deformation opportunities generated by multiple polymers used to form micro or nanofibers. Among these applications, hemostatic activity of micro and nanofiber materials is receiving more and more attention in biomedical applications. It is important to find both biomaterials and micro and nanofiber structural properties that affect organic reactions. Studies have also been conducted on major animal models used to assess the safety and effectiveness of hemostatic agents [57]. There are several ways to form nanofibers, such as electrospinning [58, 59], solution hollow spinning [59, 60], mechanical drawing [60, 61], centrifugal radiation [61, 62], and fiber self-assembly [62, 63]. Despite various formation methods and advantages [63, 64] electrospinning is the most widely used technology for forming laboratory and industrial-scale nanofibers. Electrospinning nano and microfibers are attractive in a variety of applications as they provide many opportunities for material development.

### Biomaterials for hemostatic bleeding

Interruption of bleeding hemostasis is clinically most important in prevention, surgery, and emergency scenarios. Hemorrhagic complications caused by surgery, as well as congenital, disease-related or drug-induced blood disorder, can cause serious morbidity and mortality in civilians and soldiers. In the case of externally accessible injuries, various natural and synthetic biomaterials went through strong research and led to hemostatic technologies including adhesives, bandages, tamponades, hemostatic bands, dressing and coagulation promoting powders. On the other hand, the treatment of incompressible internal bleeding still relies heavily on whole blood or hemostatic components of blood. Platelet transfusions pose significant issues of limited availability, contamination risk, low portability, performance variability, and immunological adverse events, while the use of fibrinogen or coagulation factors provides only partial mechanisms for hemostasis [64, 65].

### Thrombin coating cross-linked chitosan film

In the research, the authors intend to develop a new cross-linked chitosan film with hemostatic ability to apply sheets to patch-type hemostatic agents, and develop a new concept complex hemostatic pad that combines it with a powerful hydrocolloid pad and an adhesive [65, 66]. First, EDC/genipin (GP)/glutaldehyde (GTA) was considered as the crosslinking agent. GTA was excluded because toxicity above the standard was detected in the toxicity test. Second, an EDC/NHS-based crosslinked chitosan film was prepared [66–68, 67, 68, 69]. Crosslinking agents used in the preparation of polymer and crosslinked chitosan films were prepared using GP as crosslinking agents for the base of thrombin coating films [69, 70]. Composite hemostatic pads were evaluated through hemostatic time performance evaluation through animals, and crosslinked chitosan complex hemostatic pads were later tested with mass-produced checkpoints. Among the current hemostatic agents, adhesives or gauze/pads have always been required to use adhesive tapes, and conventional adhesive tapes have side effects after hemostasis and thus solvent-type tapes (acrylic or hot melt) are used. This can minimize patient discomfort and side effects while maintaining hemostatic performance.

## Summary and conclusion

The recent development of hemostatic biomaterials has been included in biomedical application. First, the historical background of artificial hemostasis is included. Second, it is included in more detail, focusing on the current hemostasis research. Current research on hemostasis also included oxidized cellulose-based hemostatic biomaterials such as starch based on a complex crosslinked hemostatic network, hemostatic materials in NHS-ester, hemostatic agents in local materials, and biomaterials for hemostatic management. Polysaccharide hemostatic materials, bio-inspired adhesive catechol-binding chitosan, mesoporous silica and bioactive glass for hemostatic improvement, minimally invasive hemostatic biological materials, and chitosan-based materials for hemostatic application were included. Biological properties of synthetic hemostatic agents based on natural hemostatic agents and gelatin-microbial transglutaminase mixtures were also included. Future studies should modularize hemostatic imitation and enhancement mechanisms of various materials and technologies to find the optimal system to first promote and strengthen active platelet or platelet imitation aggregation in bleeding sites. The second optionally increases the production of thrombin and fiber formation at the site. Third, the formed fibrin biopolymer network is strengthened to reduce thrombosis and amplify hemostasis. Individual components of these systems are currently under preclinical study, but these systems and potential integrated systems should study biological distribution, systemic safety, and site selective hemostasis in several well-characterized bleeding models in vivo*.*

## Data Availability

All authors accepted an availability of data and materials reported in the article. The data availability statements have been included the information on where data supporting the results reported in the article.

## Abbreviations

ADP: adenosine diphosphate
CS: chitosan
vWF: von Willebrand factor
GP: genipin
GTA: glutaldehyde
*N*HS: *N*-hydroxysuccinimide
PCS: porous composite starch
STMP: sodium tri-metaphosphate
SPC: STMP/PCS/CS
WAR: water absorption ratio
SR: swelling ratio
SEM: scanning electron microscopy
PECs: polyelectrolyte complexes
GAG: glycosaminoglycan
PEG: poly-ethyleneglycol
POx: poly (2-oxazolines)
MIS: Minimally invasive surgery
SAE: sucrose allyl ether
mTGase: microbial transglutaminase.
